# Anti-Inflammatory Effects of Adult Stem Cells in Sustained Lung Injury: A Comparative Study

**DOI:** 10.1371/journal.pone.0069299

**Published:** 2013-08-01

**Authors:** Yuben Moodley, Vijesh Vaghjiani, James Chan, Svetlana Baltic, Marisa Ryan, Jorge Tchongue, Chrishan S. Samuel, Padma Murthi, Ornella Parolini, Ursula Manuelpillai

**Affiliations:** 1 School of Medicine and Pharmacology, University of Western Australia and Royal Perth Hospital, Perth, Western Australia, Australia; 2 Department of Respiratory and Sleep Medicine, Royal Perth Hospital, Perth, Australia; 3 Lung Institute of Western Australia, Perth, Australia; 4 Center for Reproduction and Development, Monash Institute of Medical Research, Monash University, Clayton, Victoria, Australia; 5 Center for Inflammatory Diseases, Department of Medicine, Monash University, Clayton, Victoria, Australia; 6 Department of Pharmacology, Monash University, Clayton, Victoria, Australia; 7 Florey Neurosciences Institute and Department of Biochemistry & Molecular Biology, University of Melbourne, Parkville, Victoria, Australia; 8 Department of Obstetrics and Gynecology, University of Melbourne and Pregnancy Research Center, Department of Perinatal Medicine, Royal Women's Hospital, Parkville, Victoria, Australia; 9 Centro di Ricerca E. Menni, Fondazione Poliambulanza–Istituto Ospedaliero, Brescia, Italy; National Institutes of Health, United States of America

## Abstract

Lung diseases are a major cause of global morbidity and mortality that are treated with limited efficacy. Recently stem cell therapies have been shown to effectively treat animal models of lung disease. However, there are limitations to the translation of these cell therapies to clinical disease. Studies have shown that delayed treatment of animal models does not improve outcomes and that the models do not reflect the repeated injury that is present in most lung diseases. We tested the efficacy of amnion mesenchymal stem cells (AM-MSC), bone marrow MSC (BM-MSC) and human amniotic epithelial cells (hAEC) in C57BL/6 mice using a repeat dose bleomycin-induced model of lung injury that better reflects the repeat injury seen in lung diseases. The dual bleomycin dose led to significantly higher levels of inflammation and fibrosis in the mouse lung compared to a single bleomycin dose. Intravenously infused stem cells were present in the lung in similar numbers at days 7 and 21 post cell injection. In addition, stem cell injection resulted in a significant decrease in inflammatory cell infiltrate and a reduction in IL-1 (AM-MSC), IL-6 (AM-MSC, BM-MSC, hAEC) and TNF-α (AM-MSC). The only trophic factor tested that increased following stem cell injection was IL-1RA (AM-MSC). IL-1RA levels may be modulated by GM-CSF produced by AM-MSC. Furthermore, only AM-MSC reduced collagen deposition and increased MMP-9 activity in the lung although there was a reduction of the pro-fibrogenic cytokine TGF-β following BM-MSC, AM-MSC and hAEC treatment. Therefore, AM-MSC may be more effective in reducing injury following delayed injection in the setting of repeated lung injury.

## Introduction

Lung diseases such as chronic obstructive pulmonary disease (COPD) and lung fibrosis are a major cause of global morbidity and mortality. Therapeutic options that are available currently including bronchodilators and immunosuppressive agents do not significantly change the chronic course of these diseases. This is in part due to the limited ability of these agents to attenuate the continuing injury and death of structural cells that are essential for gas exchange.

Recently, adult stem cell-based therapies for lung diseases have demonstrated potential benefits in animal models. These models range from bleomycin, oleic acid and ventilator-induced lung injury [1] [2],[3],[4]. Mesenchymal stem cells (MSC) from bone marrow (BM-MSC) and umbilical cord (UC-MSC) have been shown to reduce inflammation and fibrosis in bleomycin induced lung injury [1], [5]. Although the exact mechanisms exerted by these cells remain largely unknown, immunomodulation at the sites of injury is believed to play a major role. In LPS stimulated injury, keratinocyte growth factor (KGF) from BM-MSC was found to reduce inflammation in explanted human lung tissue [6]. In addition to KGF, tumor necrosis factor alpha inducible gene-6 (TSG-6) [7] and interleukin-1 receptor antagonist (IL-1RA) are among the growing list of molecules that have been shown to mediate the anti-inflammatory functions of BM-MSC [8].

Adult stem cells derived from the fetal membranes attached to the human placenta have also been shown to improve lung injury in animal models. Cargnoni *et al* demonstrated that fetal membrane derived cells administered either trans-tracheally or intra-peritoneally 15 min post-bleomycin reduced neutrophil infiltration and fibrosis [9]. This study used a 1∶1 mixed population of mesenchymal and epithelial cells derived from amniotic and chorionic membranes [9]. As an extension of this work, studies fractionating amniotic membrane derived cells have been investigated. We demonstrated that human amniotic epithelial cells (hAEC) reduced pro-inflammatory and pro-fibrogenic cytokines, increased matrix metallo-proteinase (MMP) function while reducing tissue inhibitors of MMP (TIMP) and thereby promoting a collagen degrading environment in the injured lung [10]. Other studies investigating hAEC have reproduced similar outcomes and found improvements in lung function of bleomycin-treated mice as well as abrogation of lung injury in ventilated sheep [11] [12].

However, there are significant limitations to the translation of these cell therapies to clinical disease. Ortiz *et al* has shown that delayed injection beyond 24 h from bleomycin exposure is not as effective as early cell injection [5]. This poses considerable problems in a clinical setting since most patients are diagnosed and treated well after the onset of disease. In addition, the models themselves may not reflect the repeated injury as occurs in human lung disease such as COPD and lung fibrosis. The bleomycin-induced model of lung injury is well characterized and following a single dose that may be administered *via* several possible routes, causes acute inflammation followed by fibrosis [13]. Importantly, recent studies testing the effects of a repeat bleomycin injection have shown that the mice develop symptoms that closely resemble the clinical scenario [14]. Prior to clinical use MSC from bone marrow and amnion are routinely expanded. Thus, it would be important to compare the efficacy of expanded placental MSC and BM-MSC in a clinically relevant model. The anti-inflammatory effects of hAEC have so far been reported from animal models that have tested the primary cells [9], [10], [11], [12]. However, a recent *in vitro* study has shown that expanded hAEC retain some anti-inflammatory properties [15]. Therefore, we tested the efficacy of primary and expanded hAEC, amniotic membrane MSC (AM-MSC) along with BM-MSC, in a repeat dose bleomycin-induced model of lung injury in C57BL/6 mice and found that AM-MSC were more effective in reducing inflammation and collagen deposition compared with BM-MSC or hAEC.

## Materials and Methods

### Ethics statement

Amniotic membranes were obtained from women delivered by caesarean section at term following a normal singleton pregnancy (n = 12). Informed written consent was obtained from each participant prior to caesarean section. The isolation of AM-MSC and hAEC and study were approved by the Human Research Ethics Committees of Monash Medical Centre and Royal Women's Hospital (07411C and 05–07). Bone marrow aspirates were collected after informed written consent given by adult donors (n = 4). Bone marrow aspiration and BM-MSC isolation were approved by the Human Research Ethics Committee of Monash Medical Centre (09074A). Mice studies were approved by the Animal Research Ethics Committee of Monash University (MMCA/2008/17).

### Cell isolation and culture

hAEC and AM-MSC were isolated using protocols described previously [16], [17] with minor modifications. Purity of hAEC was assessed by flow cytometry for cytokeratins 7, 8/18. Batches with >97% cytokeratin positive cells and having typical cobblestone epithelial morphology in primary culture (passage 0 =  P0) were used in experiments described below. hAEC were also serially expanded in DMEM/F12 supplemented with 10% fetal calf serum (FCS) and 10 ng/ml of EGF to reach passage 5 (P5) [15]. P5 hAEC were also tested in experiments described below. Reagents and medium were purchased from Gibco (Grand Island, NY).

AM-MSC and BM-MSC adhered on to plastic and had a typical fibroblast appearance. MSC from amniotic membrane and bone marrow were serially expanded in DMEM/F12 with 10% FCS and P5 cells were used for cell transplantation.

Stemness and phenotypic markers used to characterize the cells are shown in Table S1.

### Cell injection into mice

Eight week old female C57BL/6 mice were given two doses of bleomycin-sulphate (0.15 mg in 20 μl saline; Sigma-Aldrich, St Louis, MO) to induce inflammation and fibrosis [1]. The first bleomycin dose was administered intra-nasally under weak anaesthesia (isoflurane; Baxter Healthcare, Toongabbie, New South Wales, Australia), and the second dose given seven days later. hAEC and AM-MSC from n = 6 randomly selected amniotic membranes and BM-MSC from n = 4 donors were pooled. One million pooled BM-MSC, AM-MSC, P0 and P5 hAEC were administered *via* the tail vein 72 h following the second bleomycin dose. The control groups consisted of healthy mice instilled with 20 µl saline and mice given the two bleomycin doses, only. Each control and experimental cohort consisted of n = 8 mice. Animals were culled one and three weeks after cell injection. Lungs were collected processed for immunohistochemistry and stored at −80°C.

### Immunohistochemistry

Lung tissue was fixed in 10% neutral buffered formalin, processed and embedded in paraffin. Staining was carried out on dewaxed tissue sections 5 μm in thickness. Injected cells were identified using an antibody specific for human inner mitochondrial membrane protein (IMM; 1∶100, Millipore, Billerica, MA) following antigen retrieval in 0.01M citrate buffer [10], [18]. Immune cells were identified with rat anti-mouse biotinylated CD45 antibody (1∶200; BD Biosciences, San Jose, CA). Briefly, after blocking endogenous peroxidase activity in 0.6% H_2_O_2_ followed by CAS protein blocking solution (Invitrogen, Camarillo, CA), sections were incubated overnight at 4°C with diluted primary antibodies followed by biotinylated rabbit anti-mouse IgG2a for IMM (1∶300; Invitrogen). Primary antibodies were omitted from negative controls. Antibody binding was detected using ABC kit reagents (Vector Laboratories, Burlingame, CA) followed by DAB chromogen (Sigma-Aldrich). Sections were lightly counterstained with haematoxylin and mounted in DPX. The semi-quantitative inflammation and fibrosis score determined the level of inflammatory infiltrate, distortion of lung architecture and fibrosis was described previously [10], [19].

### ELISA

Frozen lung samples were homogenised in RIPA lysis buffer (50 mM Tris-HCL, 150 mM NaCl, 1 mM EDTA, 1% Triton X-100, 0.5% Tween-20, 0.1% SDS) containing protease inhibitor. Between 40–60 mg of lung tissue was homogenised in 1 ml of lysis buffer, centrifuged at 14,000×g at 4°C, supernatants collected and stored at −80°C. Murine IL-1, IL-6, TNF-α and TGF-β protein content in the tissue lysates were measured according to manufacturer's recommendations. ELISA for IL-1, IL-6 and TNF-α were purchased from Life Research (Burwood East, Victoria, Australia) and TGF-β from R&D Systems (Minneapolis, MN). Samples were diluted 1∶3 for IL-1 and 1∶10 for the other assays.

GM-CSF produced by the placental stem cells and BM-MSC was also measured. Cells were grown in normal medium for 4–5 days until confluent, rinsed thoroughly in PBS and cultured in serum free medium for 48 h. GM-CSF in the conditioned medium samples was measured by ELISA (R&D Systems).

Each sample was assayed in duplicate. ELISA plates were read at 450 nm using a plate reader and concentrations calculated from the standard curves. The results were normalised to the total protein contents that were measured using the BCA assay (Thermo Scientific, Rockford, IL).

### RT-qPCR

RNA was isolated from mouse lung tissue using QIAShredders and RNeasy Mini Kit (Qiagen, Hilden, Germany) followed by RNase-Free DNase treatment to remove contaminating genomic DNA. The resulting total RNA was converted into cDNA and amplified using the QuantiTect Probe One Step RT-PCR Kit (Qiagen) with gene specific TaqMan primer probes (Applied Biosystems, Warrington, UK) to identify TSG-6, IL-1RA, KGF and GAPDH (housekeeping) gene expression. Target and housekeeping genes were amplified in the same reaction. RT-qPCR was performed according to the manufacturer's instructions on a StepOne Plus instrument (Applied Biosystems). The cDNA was synthesized at 50°C (20 min). Thereafter, the reaction mix was heated to 95°C (15 min) to inactivate the reverse transcriptase and denature the cDNA. The cDNA was amplified by PCR using the following cycling parameters: denaturation at 95°C (1 s) and anneal-extension at 60°C (60 s) for 45 cycles. Each sample was analysed in duplicate and normalized against the GAPDH housekeeping gene values. The relative ΔΔC_T_ quantitation analysis was performed to obtain a comparative concentration where healthy mice served as the internal control  = 1.

### Hydroxyproline assay

Frozen lung tissues were assessed for hydroxyproline content, as described previously [20]. Briefly, tissues were lyophilized to dry weight, hydrolysed in 6 M HCl and reconstituted in 0.1 M HCl for hydroxyproline determination. The absorbance of each sample was measured at 558 nm using a Beckman DU-64 spectrophotometer (Beckman Coulter Inc, Brea, CA) and hydroxyproline content determined from a standard curve of trans-L-hydroxy-L-proline (Sigma-Aldrich). The hydroxyproline content was divided by the dry weight of tissue and expressed as µg of hydroxyproline/mg of tissue.

### Gelatin zymography

Frozen lung tissues were also used for the assessment of MMP activity, as described earlier [21]. Equivalent amounts of protein (15 µg/sample) were loaded onto zymogram gels containing 7.5% acrylamide and 1 mg/ml gelatin and assessed for changes in MMP-2, MMP-9 and MMP-13. Gelatinolytic activity indicated by clear bands on the gels was quantified by densitometry (GS710 Calibrated Imaging Densitometer and Quantity-One software, Bio-Rad, Hercules, CA). The mean densitometry data from saline-treated, healthy control mice were expressed as 1. Data from each of the experimental groups were expressed as the relative mean ± SEM to healthy mice.

### Statistics

Data are expressed as the mean ± SEM and analysed by one-way ANOVA followed by Tukey's post hoc test. GraphPad Prism software (v5.03) was used for data analysis. P<0.05 was considered to be statistically significant.

## Results

### Dual bleomycin dose leads to significant lung injury

**Figure 1:**
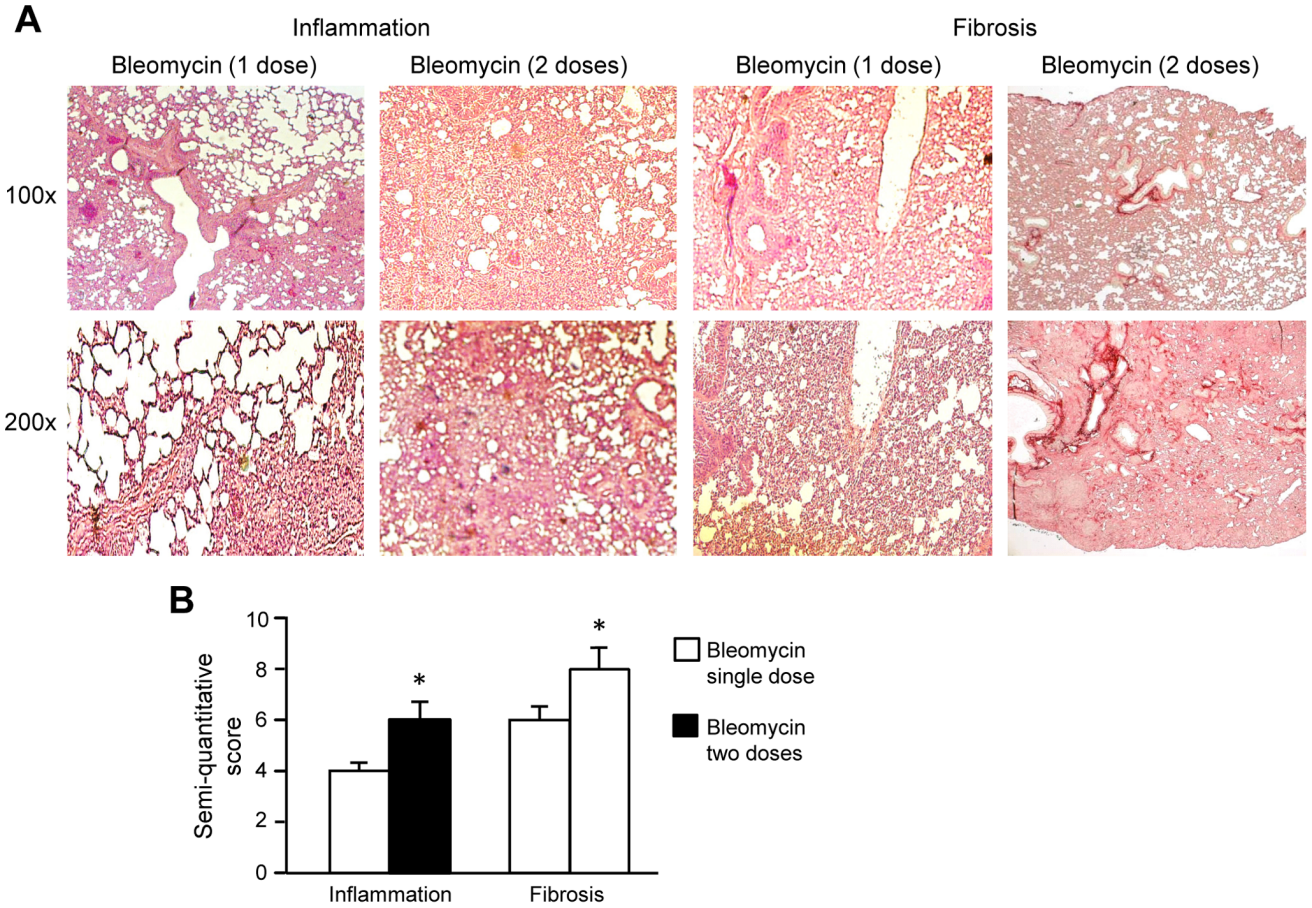
A comparison of a single versus two doses of bleomycin on lung injury in C57BL/6 mice. There was more inflammatory infiltration at day 10 and fibrosis at day 21 post-bleomycin for double versus a single dose of bleomycin (**A**). Semi-quantitative measures of inflammatory cell infiltration and fibrosis showed significant elevation of inflammation at day 10 and fibrosis at day 21, following two bleomycin doses compared to a single dose (**B**). * p<0.05.

**Figure 2:**
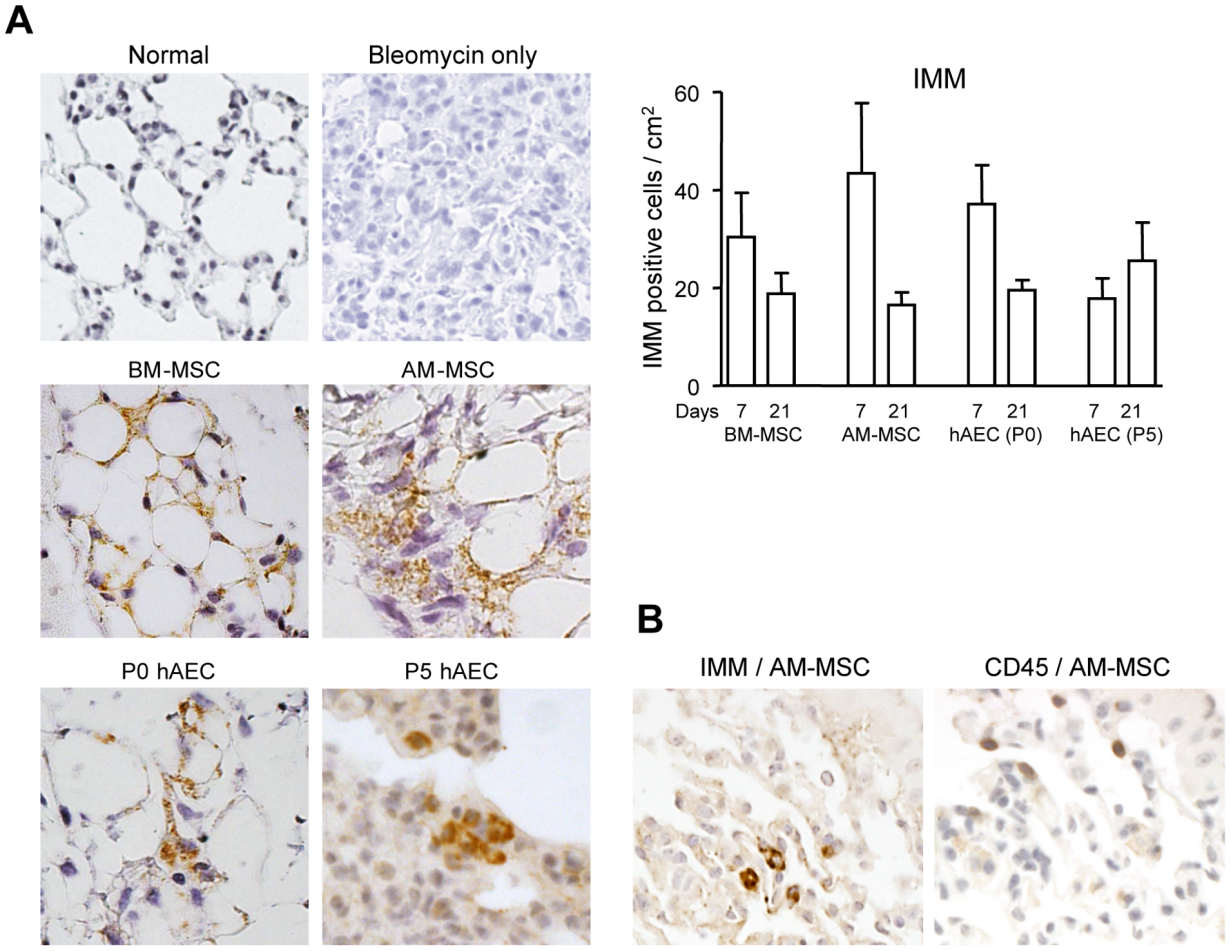
Localization of human cells in the mouse lung. Cells were infused 3 days after two bleomycin doses into C57BL/6 mice. Lung tissues were collected 7 and 21 days post cell infusion. Infused cells were localized by immunohistochemistry for human inner mitochondrial membrane (IMM) protein. The micrographs depict IMM positive cells in lungs 7 days post cell infusion. Magnification 200×. The graph depicts the number of human cells found in the lung at 7 and 21 days post cell infusion (n = 6–8 mice/cohort). No significant differences were found (**A**). Serial lung tissue sections stained for IMM and CD45 show IMM positive cells lacking CD45 staining (**B**).

Studies have shown that stem cells reduce fibrosis induced by a single bleomycin dose when injected within 24 h of bleomycin injury [5]. We examined the effects of stem cells in a clinically more relevant model of repeated lung injury where mice were instilled intra-nasally with two doses of 0.15 mg bleomycin and cells injected 3 days later. Initially, we compared the effects of a single and double bleomycin dose. In the single dose model, inflammation was maximal at daypost-second dose of the10–14 and fibrosis at daypost-second dose of the21–28. Therefore, we compared inflammation of the single bleomycin dose model at day 10 with day 10 post-second dose of the double bleomycin dose model. Similarly, we compared fibrosis at day 21. The single and dual bleomycin doses caused significant lung injury (Figure 1). Inflammatory cell infiltrates and distortion of the lung parenchyma were seen. The semi-quantitative inflammation score, was higher in mice given two bleomycin doses compared to the single dose (4±0.6 versus 6±0.6, p<0.05). The fibrosis score was also higher in mice given two doses compared to the single dose (6±0.6 versus 8±0.9, p<0.05). We then injected BM-MSC, AM-MSC, P0 and P5 hAEC into the tail vein 72 h after the second bleomycin dose. Firstly, we examined whether the infused human cells remained in the lungs. Using the human specific IMM antibody, we found that there were similar cell numbers/cm^2^ of lung tissue for all the cell types injected 7 days after cell infusion (Figure 2A). Lesser numbers of IMM positive cells were found 21 days post cell infusion. To determine whether the human cells had been phagocytosed, we examined serial sections for IMM and the common leucocyte antigen CD45. IMM positive cells lacking CD45 were seen in lung tissue sections (Figure 2B).

### Inflammation declined with cell infusion

**Figure 3:**
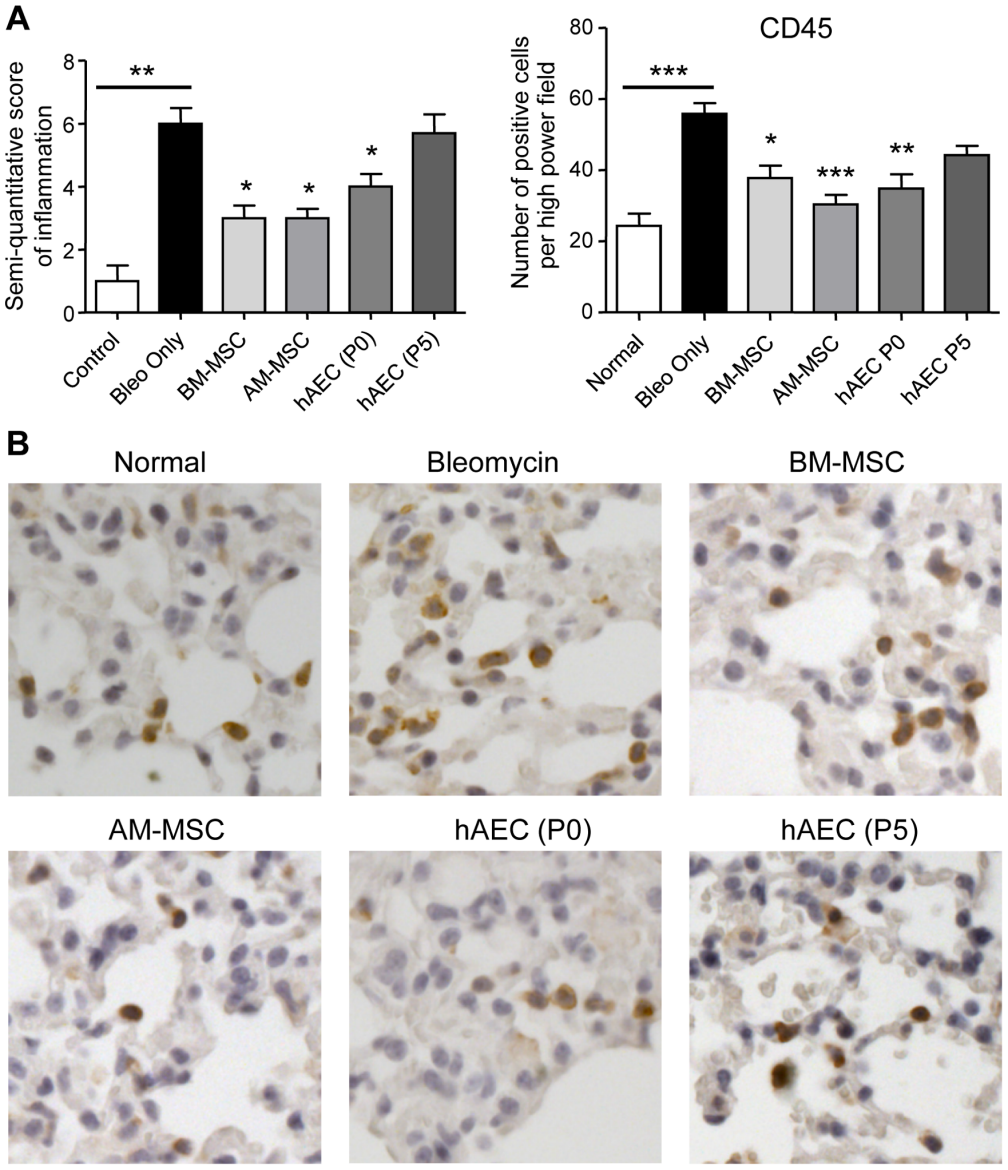
Inflammatory cells in lung tissue. C57BL/6 mice were given two bleomycin doses followed by cells and culled 7 days later. The score of inflammation was significantly elevated in bleomycin treated mice. Inflammatory infiltrates were lower in mice treated with AM-MSC, BM-MSC and P0 hAEC (**A**). Histological panels show CD45 stained leucocytes in lung tissue sections. The graph depicts the numbers of CD45+ cells. CD45+ leucocytes were reduced significantly following stem cell treatment (**B**). * and *** p<0.05 and 0.001, respectively. Each cohort analysed consisted of n = 8 mice.

Having found that stem cells were present in the lung, we examined whether there was an effect on inflammation. Semi-quantitative scoring showed that mice given bleomycin had an increased inflammatory cell infiltrate (p<0.01 for mice culled 10 days after the second bleomycin dose compared to healthy). In contrast, bleomycin treated mice given BM-MSC, AM-MSC and P0 hAEC showed a reduction in cellular infiltration (p<0.05 compared to bleomycin alone; Figure 3A). Furthermore, the reduction in the score of inflammation largely paralleled the changes in CD45+ leucocytes. CD45 stained cells in lung tissues are shown in Figure 3B. Compared to bleomycin treated mice, there was a reduction in CD45+ leucocytes in the lung following cell injection (p<0.05 for BM-MSC, p<0.01 for P0 hAEC and p<0.001 for AM-MSC; Figure 3B).

**Figure 4:**
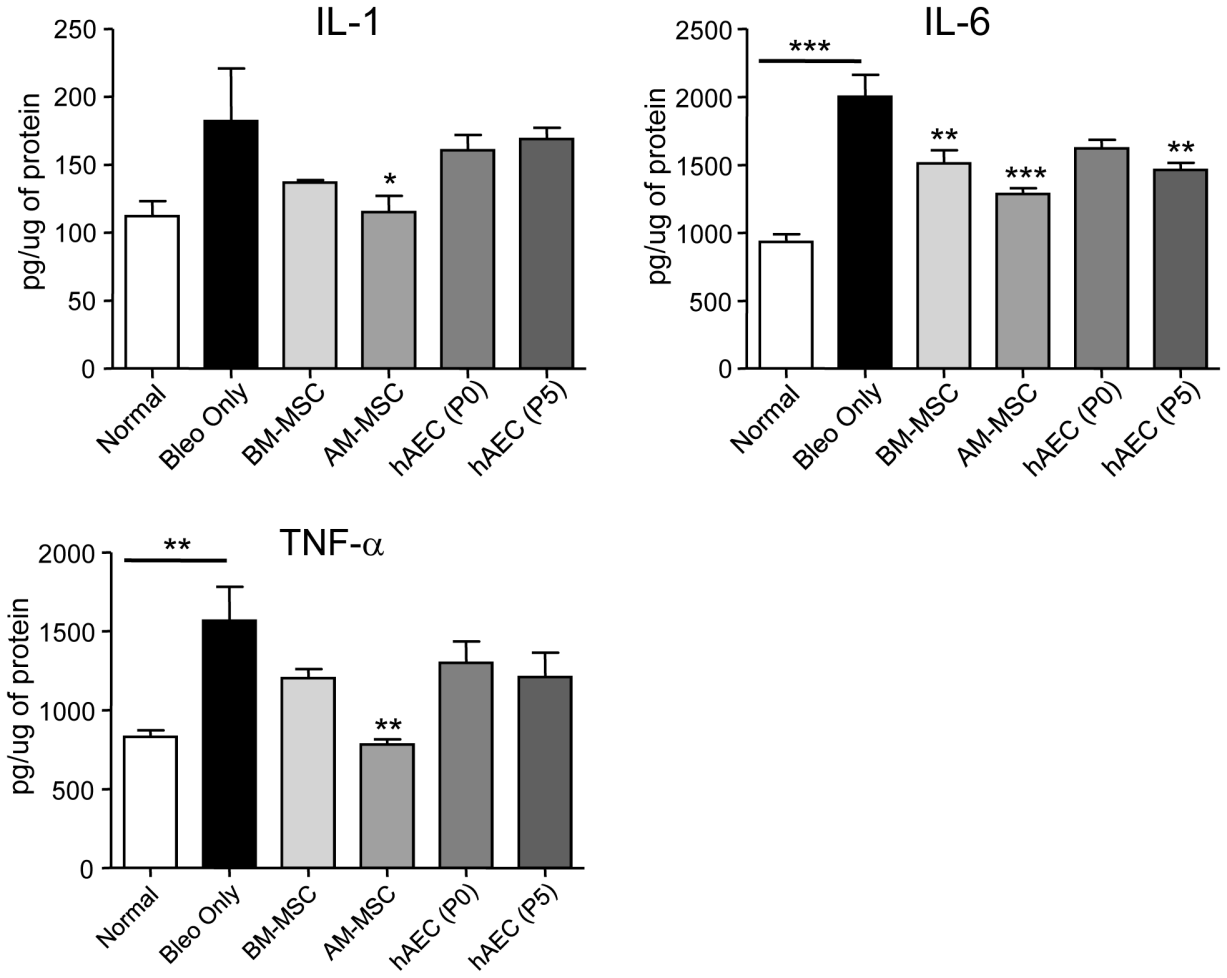
Cytokine protein levels in mouse lung tissue. C57BL/6 mice were given two doses of bleomycin followed by cells and culled 7 days later. Lung tissue lysates were analysed by ELISA (n = 8 for each cohort). TNF-α and IL-6 protein were significantly elevated in bleomycin treated mice compared to healthy controls. IL-1, IL-6 and TNF-α were reduced in mice given amnion MSC (AM-MSC) compared to bleomycin treated mice. *, ** and ** p<0.05, 0.01 and 0.001, respectively by ANOVA followed by Tukey's post hoc test.

We then assessed whether the reduction in inflammatory cell numbers were reflected in the pro-inflammatory cytokine levels by ELISA analysis of whole lung tissue lysates. Studies have shown that IL-6, IL-1 and TNF-α are elevated following a single bleomycin dose [10]. Following two bleomycin doses, these cytokines were again elevated with TNF-α and IL-6 being significantly increased (p<0.01 and 0.001, respectively compared to healthy mice; Figure 4). Compared to the bleomycin controls, IL-6 was reduced following injection of BM-MSC and P5 hAEC (p<0.01) and AM-MSC (p<0.001), while IL-1 and TNF-α were also reduced with AM-MSC treatment only (p<0.05 and p<0.01, respectively).

### Interleukin-1 receptor antagonist was raised following AM-MSC treatment

**Figure 5:**
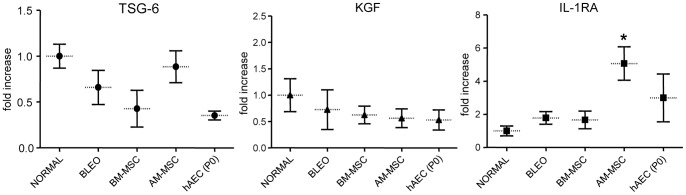
mRNA expression in mouse lung tissue. Expression in lung tissue was measured by RT-qPCR and expressed as ΔΔCt values (n = 4 for each cohort). There were no changes in TSG-6, KGF or IL-1RA mRNA expression in mice treated with bleomycin compared to healthy mice. Mice given amnion MSC (AM-MSC) showed a small increase in IL-1RA expression. * p<0.05.

Noting the fall in inflammatory infiltrate and cytokines, we investigated possible mediators of the anti-inflammatory functions of stem cells. We examined the mRNA expression of TSG-6, KGF and IL-1RA in total RNA from whole lung tissues by quantitative PCR. TSG-6 showed a fall in the bleomycin treated mice versus normal mice but was not significantly lower (Figure 5). There was no difference in TSG-6 and KGF between healthy, bleomycin only and stem cell treated mice. Notably, the only change observed was an increase in IL-1RA expression in AM-MSC treated mice (p<0.05 compared to bleomycin only; Figure 5). GM-CSF has been shown to elevate IL-1RA expression [22]. We explored whether GM-CSF from the cells could contribute to the increased levels of IL-1RA in lung tissue. Analysis of conditioned media samples showed that only AM-MSC secreted GM-CSF ( = 44±16 pg/ml).

### AM-MSC reduced established lung fibrosis

**Figure 6:**
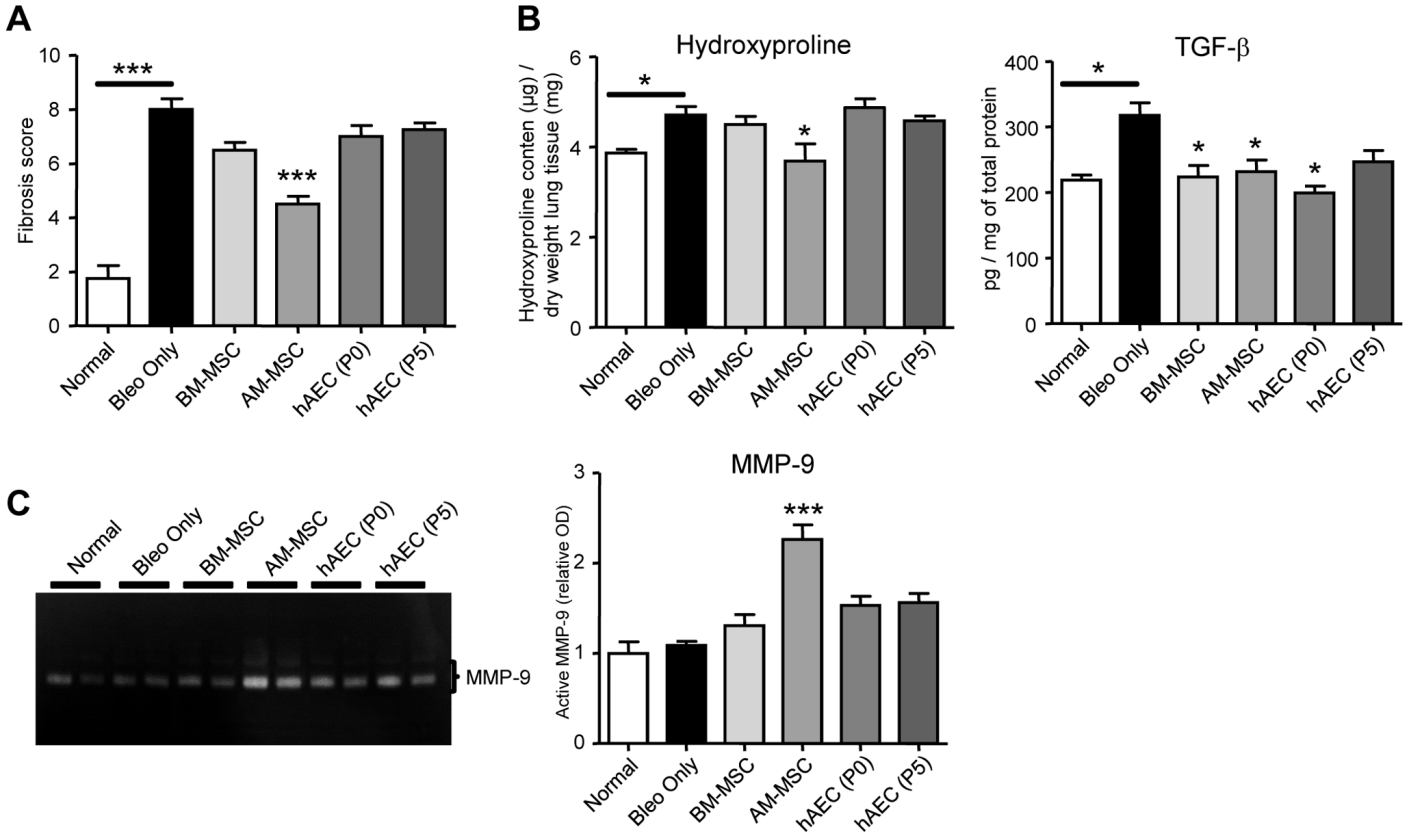
Fibrosis indices in the mouse lung. Bleomycin treated mice given mesenchymal stem cells from human marrow (BM-MSC) and amnion (AM-MSC) and examined 21 days after treatment had a reduced score of fibrosis (**A**). Hydroxyproline content (a measure of collagen levels) from each of the groups studied is also shown and was normalised to the dry weight of lung tissue. Only mice treated with AM-MSC showed a significant reduction in collagen content (**B**). Levels of TGF-β, a pro-fibrogenic cytokine, declined following cell treatment (**B**). Gelatin zymography for MMP-9 showed increased gelatinase activity in mice infused with AM-MSC compared to the other groups studied (**C**). *, ** P<0.05 and 0.01, respectively. Samples from n = 8 mice from each group were analysed.

Next, we determined if the reduction in inflammation observed after a week of cell infusion led to an attenuation of collagen deposition by examining lung tissue taken from mice culled 2 weeks later. The semi-quantitative score measuring fibrosis was increased following bleomycin (p<0.001 compared to healthy) and was lower only in mice treated with AM-MSC (p<0.001 compared to bleomycin; Figure 6A). We then examined the lung for aberrant collagen concentration. There was a significant increase in lung collagen measured by the hydroxyproline assay following bleomycin treatment when compared to normal mice (p<0.05; Figure 6B). Again, there was only a fall in lung hydroxyproline content in mice given AM-MSC (p<0.05 versus bleomycin). On the other hand, there was no change in aberrant lung collagen deposition following treatment with BM-MSC, P0 or P5 hAEC. TGF-β is a major pro-fibrotic cytokine and was measured by ELISA on whole lung tissue lysates. Lung TGF-β levels were raised following bleomycin exposure, in keeping with the increased collagen concentration found in this cohort (p<0.05 compared to normal mice; Figure 6B). Interestingly, there was a fall in TGF-β levels following treatment with BM-MSC, AM-MSC and P0 hAEC (p<0.05 for these cell types compared to bleomycin treated controls). We also investigated whether changes in MMP-2, MMP-9 and MMP-13 contributed towards reduced collagen in the lung. Increased MMP-9 gelatinase activity was seen in lung tissue of mice treated with AM-MSC compared to bleomycin controls (p<0.001; Figure 6C). MMP-2 and MMP-13 were also elevated in mice treated with cells but no significant differences were seen between the bleomycin control vs cell-treated groups (data not shown).

## Discussion

We have demonstrated for the first time that adult stem cells can reduce inflammation in a murine model of repeated lung injury. The major challenge in adult stem cell therapy is to determine whether stem cells would be effective in repeated lung injury that is present in respiratory disorders such as Acute Respiratory Distress Syndrome (ARDS) where there is repetitive ventilator-induced lung injury. In progressive conditions there is acute inflammation in the background of ongoing inflammation as occurs with acute exacerbations of COPD. Notably, the vast majority of studies have examined the role of stem cells following acute injury and studies examining cell therapy during repeated lung injury is limited. [22] [23], [24]. We therefore modelled this clinical scenario in a double dose bleomycin-induced model of lung injury. Although the model does not represent all aspects of human lung disease, it has salient characteristics that represent major features of lung pathophysiology. The first dose of bleomycin causes inflammation and the fibrotic process would commence around day 7 when the second dose of bleomycin was administered. This second dose would cause further injury on the background of ongoing inflammation and early fibrosis. Brown *et al* demonstrated that lymphocytes and polymorphonuclear cells were present at 28 days and persistent macrophage infiltration up to 60 days following 2 doses of bleomycin [25]. In addition they showed pulmonary oedema was present at 28 days and that there was persistence of fibrosis at 90 days after the second dose of bleomycin [25]. Furthermore, Pinart *et al* has also shown that a double dose of bleomycin mimics the mechanical dysfunction of the lung as occurs in chronic human interstitial lung disease [14].

In addition to the model, we delayed the tail vein injection of stem cells to 3 days after the second dose of bleomycin. A number of studies have demonstrated the benefit of stem cells administered within 24 h of injury but that stem cells have limited efficacy when injected more than 24 h post injury [5] [6] [10]. Little is known about the effects of delayed cell injection on lung tissue inflammation. This is clinically relevant since most patients present several days post-symptoms.

Another significant point of our study was to compare cells derived from bone marrow and amniotic membrane with each other. Several studies have shown that hAEC, BM-MSC and AM-MSC have reduced lung injury in animal models, however no direct comparison has been made between these cells in the lung [9],[26]. We found using the human specific inner mitochondrial membrane (IMM) antibody that the adult stem cells were present in the lung for at least 21 days post injection and together with CD45 staining demonstrated that at least some of these human cells had not been phagocytosed by the CD45+ leucocytes. However, since mitochondria can be transferred between cells [27], we cannot exclude that IMM+ staining could have been from transferred mitochondria. The IMM+ cell numbers found were similar between groups in keeping with several studies [1], [9] however, whether these resident cells arise due to proliferation in the lung remain unknown.

We found that the inflammatory cell infiltrate, CD45+ cells and the pro-inflammatory cytokines IL-6 and TNF-α were elevated in the bleomycin-induced lung injury. BM-MSC, AM-MSC and P0 hAEC were equally effective in reducing the numbers of inflammatory cells in the lung. This may be partly due to the suppression of chemokines such as MCP-1 and MIP-1α and cytokines regulating immune cell recruitment into the lung. Indeed, in a recent study on liver fibrosis, mice infused with P0 hAEC had reduced MCP-1 in the liver and fewer numbers of inflammatory cells [10], [18]. P5 hAEC have been reported to secrete substantial amounts of MCP-1 [15], and this may partly explain the elevated inflammatory cell infiltrate in P5 hAEC treated mice. BM-MSC, AM-MSC and hAEC reduced IL-6 protein while AM-MSC also reduced TNF-α. As both of these cytokines act synergistically to increase inflammation and fibrosis [29], the inhibition of IL-6 and TNF-α protein would be expected to significantly attenuate inflammation in AM-MSC treated mice. Interestingly, there was no increase in IL-1 between bleomycin treated mice and controls. We suggest that this may be due to different dynamics of IL-1 release following repeated bleomycin injury. However, IL-1 was reduced by AM-MSC to levels similar to healthy controls. IL-1 is known to initiate chronic inflammatory lesions and reduction of IL-1 protein by AM-MSC would further attenuate the inflammation and fibrosis.

The treatment of fibrosis by adult stem cells is proven to be more challenging. This study showed that AM-MSC was the only cell type to reduce aberrant collagen levels as measured by the hydroxyproline assay. Possible mechanisms include an AM-MSC-induced reduction in TGF-β which would reduce collagen deposition, and the significant increase in MMP-9 activity that would increase collagen breakdown [28]. TGF-β and pro-inflammatory cytokines can induce MMP-9 [29], [30]. However, given that TNFα, IL-6, IL-1 and TGF-β protein levels were reduced with AM-MSC treatment, other factors such as BMP-7 that can down-regulate TGF-β in the lung and induce MMP may play a role [31]. P21 is present in the lung and one of its isoforms Pak1, has also been shown to reduce TGF-β expression while inducing MMP-9 [32]. Whether AM-MSC induces such pathways that can lead to anti-fibrotic effects need further investigation.

We further examined molecules that could mediate reparative properties of stem cells such as IL-1RA, KGF and TSG-6 [8], [33], [34] [27]. IL-1RA is secreted by a wide variety of cells including inflammatory and epithelial cells, and is raised during inflammation and inhibits the inflammatory actions of both IL-1a and IL-1b [35]. Ortiz *et al* has shown that IL-1RA is raised following trans-tracheal bleomycin but decreased following MSC injection [8]. We found no change in IL-1RA levels with bleomycin treatment but that expression was raised by AM-MSC. The AM-MSC induced increase in IL-1RA would be anti-inflammatory and provide another explanation for the actions of AM-MSC. This observation is in line with a study that demonstrated IL-1RA released from amniotic mesenchymal stem cells reduced hepatic injury in mice [36]. Furthermore, we have shown that AM-MSC secrete GM-CSF unlike the other stem cells studied. Since GM-CSF has been shown to stimulate the release of IL-1RA, it may partly explain the increase in IL-1RA expression [37]. KGF is secreted by fibroblasts and MSC, is a potent mitogen for epithelial cells and is critical in the development of the lung [38]. However, we did not find any changes in KGF mRNA expression in our studies. TSG-6 is not expressed constitutively in adult lung tissue but is secreted by many types of cells in response to inflammation and is raised in a variety of different inflammatory conditions [39]. In our study we found no changes in TSG-6 in bleomycin or cell treated groups. TSG-6 may not have a sustained increase in expression in this model in contrast to the acute models of injury reported by others.

Taken together, this study shows that adult stem cells (AM-MSC, BM-MSC and P0 hAEC) exert a wide range of anti-inflammatory effects despite delayed injection in a repeated bleomycin model of lung injury and supports their role in the treatment of inflammatory components of lung disease irrespective of chronicity. Compared with BM-MSC and hAEC, AM-MSC reduced established fibrosis by several possible mechanisms including reduced TGF-β, increased MMP-9 activity, GM-CSF secretion and induction of IL-1RA.

## Supporting Information

Table S1 **Stemness and Phenotypic Markers.**
(DOCX)
