# Supplementary material for: Anti-Inflammatory Effects of Adult Stem Cells in Sustained Lung Injury: A Comparative Study
Source: PLoS One. 2013 Aug 1;8(8):e69299. doi: 10.1371/journal.pone.0069299 (PMC3731305; doi:10.1371/journal.pone.0069299)
Supplement: Table S1 — Stemness and Phenotypic Markers. (DOCX) [file pone.0069299.s001.docx]

**Supplemental Table S1**: Stemness and Phenotypic Markers

| Markers | BM-MSC  (P5) | AM-MSC  (P0 / P5) | hAEC  (P0) | hAEC  (P5) |
| --- | --- | --- | --- | --- |
| Stemness markers^a^ |  |  |  |  |
| Oct-4 | √ | √ | √ | n.d |
| Sox-2 | √ | √ | √ | n.d |
| Nanog | √ | √ | √ | n.d |
| SSEA-4 |  | √ (P0) | √ | n.d |
|  |  |  |  |  |
| Phenotypic markers^b^ |  |  |  |  |
| Cytokeratins 8/18 | n.d | n.d | √ | 14% |
| Vimentin | √ | √ | 37% | √ |
| CD29 | √ | √ | √ | √ |
| CD73 | √ | √ | 76% | 64% |
| CD90 | √ | √ | 33% | √ |
| CD105 | √ | √ | n.d | 28% |
| CD31 | n.d | n.d | n.d | n.d |
| CD34 | n.d | n.d | n.d | n.d |
| CD45 | n.d | n.d | n.d | n.d |
| HLA-A,B,C | √ | √ | √ | √ |
| HLA-G | √ | √ | √ | 18% |
| HLA-DP, DQ,DR | n.d | n.d | n.d | n.d |

^a^ mRNA expression. ^b^ Flow cytometery with √ indicating >90% immunopositive cells; mean

values from n=3 shown if <90%. n.d = not detected.
